# Effectiveness of Nd:YAG Laser on the elimination of debris and Smear Layer. A comparative study with two different irrigation solution: EDTA and QMix® in addition to NaOCl

**DOI:** 10.4317/jced.54395

**Published:** 2018-01-01

**Authors:** Paloma Montero-Miralles, Roberto Estévez-Luaña, César DeGregorio-González, Oliver Valencia-dePablo, David E. Jaramillo, Rafael Cisneros-Cabello

**Affiliations:** 1 Universidad de Sevilla. School of Dentistry. Sevilla

## Abstract

**Background:**

The aim of this study was to evaluate the effectiveness in dentin debris and smear layer removal from root canal walls using EDTA and QMix® alone and also activated with Nd:YAG laser.

**Material and Methods:**

50 single-rooted teeth were instrumented and divided in 5 groups according to irrigation protocol: 17% EDTA, QMix®, Nd:YAG laser alone, and combination of 17% EDTA - Nd:YAG laser and QMix® - Nd:YAG laser. Samples were evaluated using SEM. Statistical analysis was done using Chi-Square Fisher exact test and McNemar test.

**Results:**

Dentinal debris analysis showed statistically significant differences when comparing 17% EDTA vs Laser and Laser vs QMix® in combination with Laser at the apical third. The Smear Layer analysis also showed statistically significant differences at the apical third when comparing 17% EDTA vs Laser, QMix® vs QMix® in combination with Laser and Laser vs QMix® in combination with Laser.

**Conclusions:**

17% EDTA was the most efficient irrigant showing the best results. Laser alone was not effective removing either dentinal debris or smear layer.

** Key words:**Laser, endodontics, Smear Layer.

## Introduction

The smear layer is a microscopic layer formed after root canal instrumentation and located along the root canal walls. It blocks dentinal tubule orifices and creates an interface between filling material and root canal wall, affecting sealing ability of the root canal system. The width of this layer is between 1 to 2 microns (1) and it reduces penetration of irrigants and sealers into dentinal tubules (2,3).

Some studies have shown that mechanical instrumentation and chemical action of NaOCl are not enough to remove the smear layer totally from the root canal wall (4,5). Chelating agents are used for its removal. QMix® (Denstply-Maillefer, Tulsa, USA) has been recently launched, composed of an antimicrobial agent, Chlorhexidine, mixed with a chelating agent, EDTA, and a surfactant (6).

Laser technology has been developing for several years and has become more widely used in the medical and dental field. Its effectiveness will depend upon such factors as: energy level, duration of exposition, absorption in tissues, root canal geometry and the distance between the tip of the laser and the tissue being treated (7-9). The most studied laser in dental literature is the Neodymium:Yttrium-Aluminium-Garnet (Nd:YAG), with a wavelength of 1064 nm and possessing partial water absorption. In endodontics, its effect on the root canal wall produce removal of smear layer and pulp tissue remnants, root canal decontamination, organic tissue vaporization inside dentinal tubules and fusion and crystallization of the inorganic component of the dentin (melting). It also produces physical-chemical changes on hydroxyapatite crystals, which modifies dentin solubility, thus becoming less susceptible to acids action.

The objective of the present study was to evaluate the cleaning capability of QMix®, 17% EDTA in combination with sodium hypochlorite, plus the action of Nd:YAG laser alone and with the previous chelating solutions.

## Material and Methods

This study was approved by the Ethical Committee of the European university of Madrid.

50 freshly extracted single-rooted human teeth were kept in 10% formalin until used. Inclusion criteria were: presence of only one root canal, complete apical closure and no previous root canal treatment done prior to its use.

The crowns of all teeth were cut off with the use of a diamond disk (Buehler, Düsseldorf, Germany) and tooth length standardized to 16 mm. A glide path was performed with a #20 hand K-Flexofile (Denstply-Maillefer, Tulsa, USA) and the cleaning and shaping of the root canal was completed using the Mtwo® rotary system (VDW, Munich, Germany) up to 40.04 to 15mm. Care was taken to keep root canal patency at all times.

During instrumentation, the root canal was irrigated in between files with 1 ml of 4.2% NaOCl using a 5 ml Monoject irrigation syringe and a 27g irrigation needle (Tyco HealthCare Group, Norwalk, CT, USA) that was kept 1 mm short of the working length. After this step, teeth were kept in saline solution until processed for SEM.

Samples were randomly divided using the software www.random.org into 5 groups of 10 teeth each. For the final irrigation cycle, all roots were sealed apically with modeling wax (Cera Reus, Reus, Spain) and a reservoir was created coronally.

Study groups.

Group 1: 1 minute of irrigation with 5 ml of 17% EDTA (Pulpdent, Oakland, MA, USA), followed by 5 ml of 4.2% NaOCl for 2 minutes and a final rinse with 2.5 ml distilled water.

Group 2: 1 minute of irrigation with 5 ml of QMix® (Denstply-Maillefer, Tulsa, OK, USA) and a final rinse with 2.5 ml distilled water.

**Figure 1:**
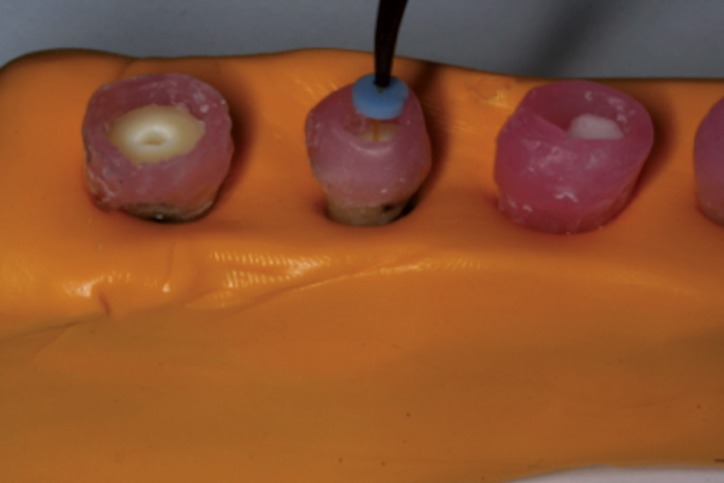
Nd:YAG laser application.

Group 3: Nd:YAG laser (DEKA, Firenze, Italy), followed by described protocol by Gutknecht and Behrens: power set at 1.5 W, 15Hz and 100 mJ of energy [10]. Wavelength of 1.064 nm. A 320 microns optic fiber was used with an apical to coronal helicoidal motion. Working length was established at 15 mm. A rubber stop was placed on the fiber optic tip at 14 mm to set this as a working field for the laser. The laser was activated on a 5 seconds cycle five times taking a rest for 20 seconds in between each cycle. 2.5 ml of distilled water was used as a final rinse (Fig. 1).

Group 4: 1 minute of irrigation with 5ml of 17% EDTA plus laser. The laser tip used as previously described; then, a rinse with 5 ml of 4.2% NaOCl for 2 minutes and a final rinse with 2.5 ml of distilled water.

Group 5: 1 minute of irrigation with 5 ml of QMix® in combination with laser, used as previously described and a final rinse with 2.5 ml of distilled water.

Roots were longitudinally split using a 20x0.25 mm fine diamond disk (Buehler, Düsseldorf, Deutschland) in a low speed motor (NSK, Japan), avoiding passing the disk through the root canal lumen to prevent the accumulation of sectioning debris. With the help of a fine chisel and with a very fine pounding, both halves of the root were obtained.

Roots were treated with a serial dilution of different concentrations of alcohol (30% to 100%) for the dehydration process. Samples were mounted on a special stainless steel base and taken inside the sputtering machine to be coated with a fine layer of graphite (Blazer Union Med 010). Immediately after, the samples received a 25 nm layer of gold (Emitech K550X). A total of 60 samples were observed under SEM. A SEM (JEOL JSM-6400) microscope was used with a 20kV, 100 mA. The working distance was set at 39 mm. A specific area of the apical and middle third of the root canal was chosen to be the observation spots.

Pictures were randomly taken first at 20X magnification for the correct localization of the observation spot. Images were centered at the apical zone in order to observe the mark left behind by the apical calibration instrument. At 0.5 mm from this spot a 100X image was taken. Two images were obtained at 500X and 1000X magnification. Magnification was established at 100x again to reach out the middle third, and two images were taken at 5 mm from the working length, at 500X y 1000X. The same procedure was done at the middle area of the root canal.

**Figure 2:**
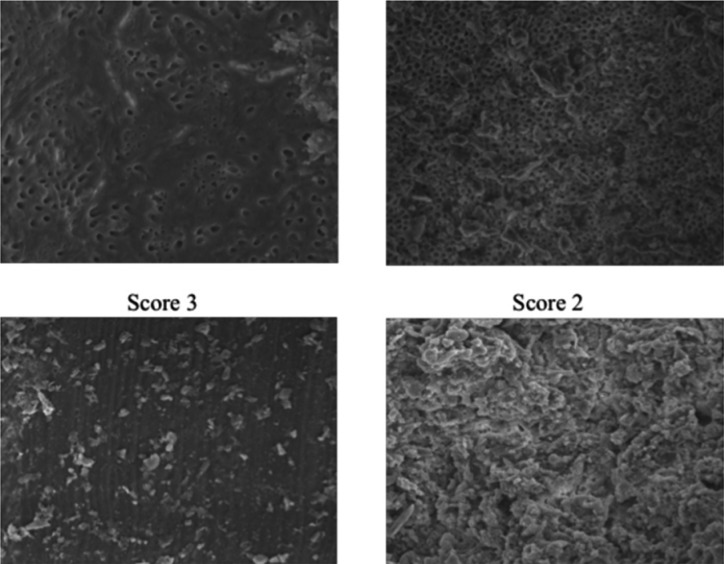
Hülsmann modified classification.

All images were analyzed by a previously calibrated expert viewer. A modified Hülsmann classification (11) was used to measure at the 500X level for the presence of dentinal debris and at 1000X for the presence of Smear Layer (Fig. 2).

The scores values was assigned in this way.

Score 0: specimens showing a clean surface and most of the dentinal tubules open

Score 1: specimens showing most of the dentinal tubules open but remaining debris covering less than a 25% of the analyzed area 

Score 2: specimens showing majority of dentinal tubules plugged with smear layer and remaining debris covering less than a 50% of the analyzed area

Score 3: specimens showing no dentinal tubules open and remaining debris covering less than a 75% of the analyzed area 

Association between all groups and the degree of cleanliness, were evaluated using the chi-square and Fisher exact test for those cases where more than 25% of the samples were less than 5. For all the tests, a signification value of 5% will be accepted. The frequency distribution was evaluated within the variable results with 2 magnifications (500X and 1000X) using the McNemar test. Data was analyzed with the SPSS 15.0 software (SPSS Inc, Chicago, IL, USA).

## Results

On dentin debris analysis, in the apical third, the best cleanliness was achieved by 17% EDTA in combination with laser and the results showed a statistically significant difference (*p*= .015) when compared with the 17% EDTA group. Laser in combination with QMix® was found better (*p*= .028) than QMix® alone. In the middle third, no statistically significant differences were found at any studied group.

The smear layer analysis, in the apical third, when comparing 17% EDTA in combination with laser (*p*= .031) with 17% EDTA, the combination of EDTA and laser showed better results and a statistically significant difference. Also, a statistically significant difference was found when comparing QMix® and QMix® in combination with laser (*p*=.029) where QMix® in combination with laser showed better results when comparing Laser in combination with QMix® vs Laser, (*p*= .03) a statistically significant difference was also found presenting QMix® in combination with laser better results. In the middle third, no statistically significant differences were found in any studied group, ( Table 1- Table 3).

**Table 1:**
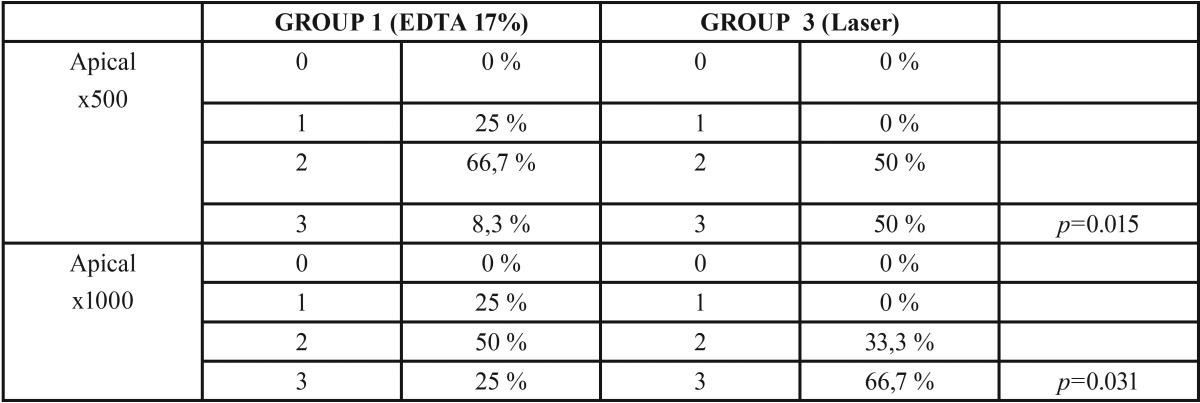
Comparative debris and Smear Layer analysis in apical third (17% EDTA and Laser).

**Table 2:**
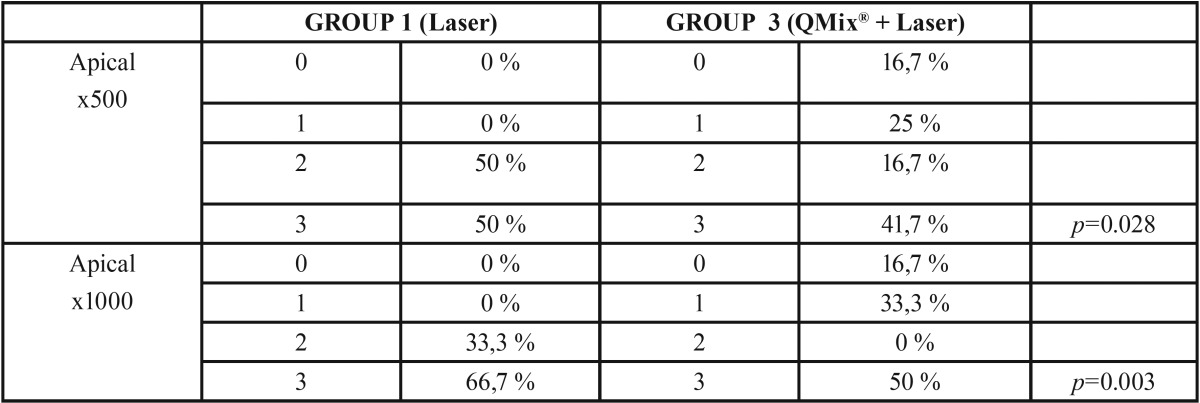
Comparative debris and Smear Layer analysis in apical third (Laser and QMix® + Laser).

**Table 3:**
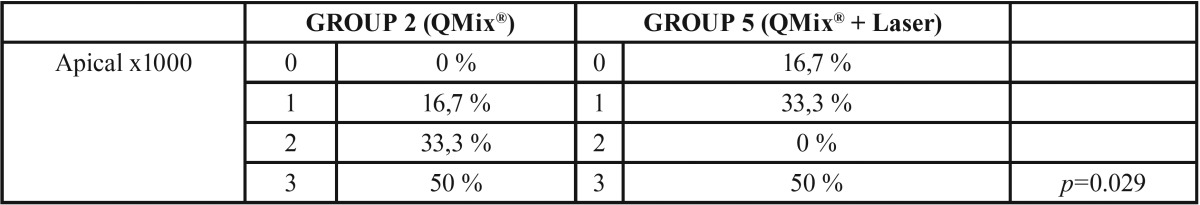
Comparative Smear Layer analysis in apical third (QMix® and QMix® + Laser).

## Discussion

The smear layer removal has been thoroughly investigated by many authors, during the last decades. The presence of this microscopic layer, could affect the adequate sealing of the root canal system. The removal of this inorganic matter, which contains some organic remnants as well, is particularly difficult in the apical third. The objective of the present study was to evaluate the cleaning ability of QMix® in combination with Nd:YAG laser compared to 17% EDTA, for the dentin debris and smear layer removal.

Root canals were prepared to 40.04 allowing the 320 microns Nd:YAG fiber laser tip to reach 1 mm short of the established working length and at the same time allow for a better flowability of the selected irrigant (12). By doing this, a size 44 diameter was made at the level where the laser tip was placed, which allowed enough space between the canal walls and this tip.

In order to simulate a clinical condition, we used modeling wax in the apex to keep the root canal system closed avoiding the extrusion of the irrigant used during the final rinse step. Dai *et al.* used an open system in their comparative study of smear layer removal using 17% EDTA and QMix® (6). They concluded that it was not possible to completely remove the smear layer. Their results showed no difference. In the present study, 17% EDTA and QMix® were not effective at apical and middle thirds. 17% EDTA was superior to QMix® at all levels. There was a statistically significant difference in dentin debris removal at middle third. At this level, QMix® was better than 17% EDTA where dentinal tubules were clean 8.3% of the time, with no statistically significant difference.

Time and concentration for the application of chelating agents is an important factor (13). Dai *et al.* applied QMix® for 2 minutes while Stojicic et al., used it for 5 minutes (14). In our study, irrigation timing was standardized at 1 minute (15-18). By doing this, it was possible to obtain better results with QMix® vs 17% EDTA for the smear layer removal, without a statistically significant difference. More studies are needed to standardize the correct application timing for QMix.

For the smear layer removal, the recommended volume for 17% EDTA is in between 3 to 20 ml per root canal (11,17). Mello *et al.* showed that a final rinse with 5ml of 17% EDTA was as effective as 10-15 ml of EDTA for smear layer removal (19). In our study, 5 ml of 17% EDTA was used in accordance with Mello´s study.

In this study the specimens under treatment with laser, showed a minimal amount of open dentinal tubules. The parameters used were 1.5 w of power, a frequency of 15Hz and 100 mJ of energy (10). 100% of QMix® in combination with laser samples, presented a score of 2-3. QMix® in combination with laser samples and laser samples showed the same results at the middle portion of the root canal. Our results are in accordance with those from Hasheminia *et al.*, Zhang *et al.*, Barbakow *et al.*, and Kivanc *et al.*. Their results showed that Nd:YAG Laser is not as effective as EDTA for the smear layer removal (20-23).

The aim of our study was to show not only if the laser was effective in the removal of the smear layer, but also, if adding a chelating agent could improve its efficacy. When the apical third was studied, the group treated with QMix® in combination with laser, showed a better cleaning ability than using QMix® alone. When the middle third was studied, dentin debris removal showed similar results in both groups, and the smear layer removal was better in the group irrigated with QMix®. This could be explained because QMix® was used only for one minute and it might need more contact time to achieve better results as proposed by Stojicic *et al.* (14).

In all groups where the laser was used, a better smear layer removal was shown. No groups treated with EDTA obtained a value of 0 (complete clean surface), but there was always a superior cleanliness at the apical third (on both smear layer and dentin debris removal) although the difference was not definitive in the middle third.

Moura-Netto *et al.* (24) found in their study that Nd:YAG laser fuses and solidifies the dentinal surface with a partial removal of dentinal debris. In agreement with this study and based on the results obtained in the present study, we can conclude that we cannot compare the use of laser with the use of chelating agents, because these solutions possess the ability to dissolve smear layer, while lasers produce melting, vaporization and crystallization of this matter.
